# Multisystem challenges in reproductive medicine: A case report of bicornuate uterus, fibroadenoma, and postpartum thyroiditis

**DOI:** 10.1097/MD.0000000000047007

**Published:** 2026-01-02

**Authors:** Fatima Faisal, Aiman Javed, Faisal Muzaffar, Tirath Patel, Ahmed Ibrahim, Fathimathul Henna, Hafiza Tooba Siddiqui, Bhumi Daishik Patel, Nana Sardarova, Nikhilesh Anand

**Affiliations:** a Department of Medicine, Dow Medical College, Karachi, Pakistan; b Department of Medicine, Dow University of Health Sciences, Karachi, Pakistan; c Department of Medicine, Multan Medical & Dental College, Multan, Pakistan; d Department of Internal Medicine, Trinity Medical Sciences University School of Medicine, Kingstown, Saint Vincent and the Grenadines; e Department of Medicine, Dow Medical College, Dow University of Health Sciences (DUHS), Karachi, Pakistan; f Department of Medicine, Dubai Medical College for Girls, Dubai, UAE; g Department of Medicine, Jinnah Sindh Medical University, Karachi, Pakistan; h Department of Internal Medicine, Windsor University School of Medicine, Saint Kitts and Nevis; i Department of Internal Medicine, Henry Ford Warren Hospital, Warren, MI; j Department of Medical Education, University of Texas Rio Grande Valley, Edinburg, TX.

**Keywords:** bicornuate uterus, hyperprolactinemia, metformin, multidisciplinary care, postpartum thyroiditis, recurrent miscarriage

## Abstract

**Rationale:**

A bicornuate uterus is an uncommon mullerian duct malformation strongly associated with recurrent early pregnancy loss and adverse obstetric outcomes. When combined with endocrine disturbances such as hyperprolactinemia and postpartum thyroid dysfunction, fertility becomes even more challenging. This case highlights how coordinated reproductive-endocrine management can overcome multiple intersecting barriers to conception and successful pregnancy.

**Patient concerns:**

A 24-year-old woman presented with a history of 3 first-trimester miscarriages, difficulty conceiving, and prior breast fibroadenoma excision. Following the birth of her first child, she later developed symptoms of palpitations, weight loss, and anxiety suggestive of thyroid dysfunction.

**Diagnoses:**

Pelvic ultrasonography identified a bicornuate uterus. Additional evaluation revealed isolated hyperprolactinemia, a benign right breast fibroadenoma, and postpartum-onset hyperthyroidism occurring several months after initiating depot medroxyprogesterone acetate (DMPA) contraception.

**Interventions:**

Management included empirical hormonal and metabolic support with progesterone, metformin, low-molecular-weight heparin, and low-dose aspirin, together with thyroid hormone therapy. She was followed jointly by obstetrics and endocrinology throughout conception, pregnancy, and the postpartum period.

**Outcomes:**

The patient achieved a viable term pregnancy and delivered a healthy infant via cesarean section at 38 weeks. Postpartum thyrotoxicosis was confirmed biochemically and successfully managed with antithyroid medication.

**Lessons:**

This case demonstrates that favorable reproductive outcomes are achievable even in the presence of significant uterine malformation and endocrine comorbidities when care is individualized and multidisciplinary. The temporal relationship between DMPA initiation and postpartum thyroiditis raises a clinically relevant question about progestin-triggered autoimmune thyroid dysfunction, warranting further study. Early imaging and endocrine profiling should be routine in women presenting with recurrent pregnancy loss.

## 1. Introduction

Congenital uterine malformations arise during Müllerian duct development and are caused by incomplete fusion or resorption of the septal canal. A malfunction in the merging of Müllerian ducts causes uterine malformation of the bicornuate uterus. Unfavorable early pregnancy and prenatal events, including recurrent miscarriages, premature labor, and birth, are linked to the rare abnormality known as the bicornuate uterus.

Two symmetrical uterine cavities that are joined caudally and have some connection between them, typically at the uterine isthmus, are features of the bicornuate uterus. A heart-shaped uterus rather than a pear-shaped one results from this abnormality.^[1]^ The most common disease among the recurrent miscarriage patients in one of the studies was endocrinological diseases, which affected 38.9% of cases.^[2]^

Hyperthyroidism is characterized by excessive thyroid hormone production. Triiodothyronine (T3) may be involved in the formation and progression of breast cancer since it can boost the effect of estradiol on cell proliferation and promote the proliferation of breast cancer cells in specific cell lines. Hyperthyroidism is substantially linked to an elevated risk of breast cancer.^[3]^

## 2. Case presentation

A 24-year-old woman presented with a complex reproductive and endocrine history. Her clinical course included 3 pregnancies, recurrent miscarriage, a surgically managed benign breast tumor, postpartum thyroid dysfunction, and a congenital uterine anomaly.

Her first pregnancy ended in spontaneous miscarriage at 6 weeks without any documented hormonal or anatomical support. An ultrasound was not done as pregnancy ended at 6 weeks, and early pelvic scan facilities were not available.

During the second pregnancy, she was administered progesterone (Cyclogest suppositories), oral dydrogesterone, and 3 weekly injections of Gravibinon. Despite this, a miscarriage occurred at 9 weeks of gestation. Two months after the second miscarriage and before her third conception, she developed a right breast fibroadenoma measuring approximately 2.5 cm in diameter. Histopathology confirmed the benign nature of the lesion. She underwent a successful lumpectomy without complications.

**Figure 1.**
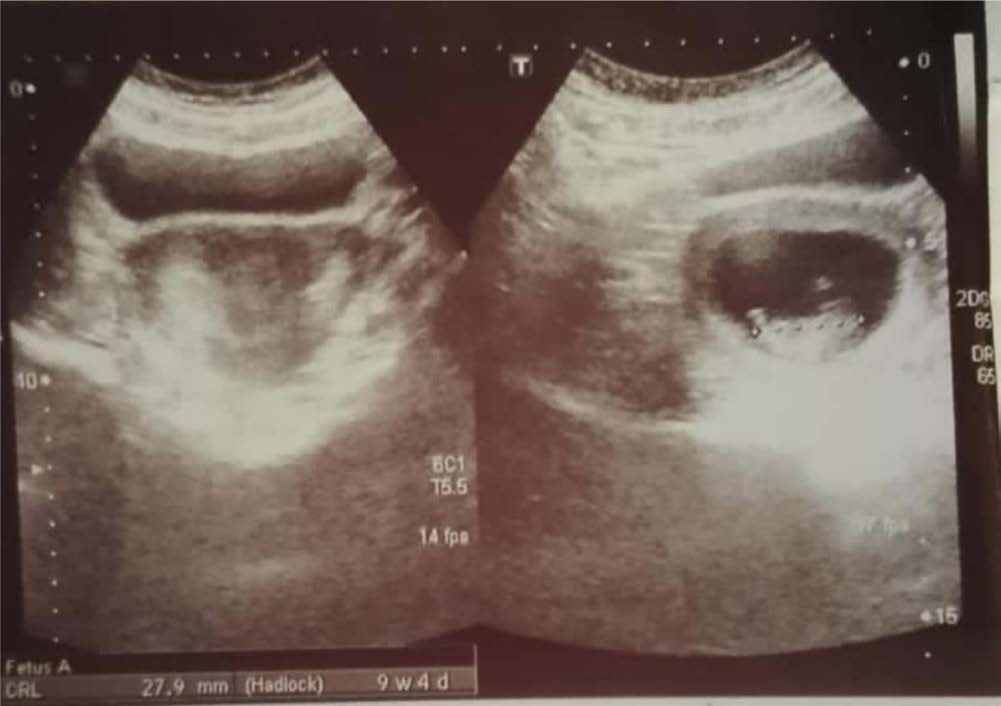
Ultrasound showing a bicornuate uterus.

Approximately 9 months later, she conceived again. A transvaginal ultrasound at 9.2 weeks confirmed a bicornuate uterus (Fig. 1), a finding not detected in previous pregnancies (Table 1). Management during this pregnancy included the following: Gravibinon, administered as 3 weekly injections until fetal cardiac activity was visualized; Pubergen (chorionic gonadotropin), continued until 17 weeks’ gestation; and Cyclogest (Progesterone), administered at 400 mg twice daily until 36 weeks, then reduced to once daily. Clexane (enoxaparin): 40 mg daily subcutaneous injections until 34 weeks because of the history of recurrent miscarriages and presence of bicornuate uterus, despite a negative thrombophilia screen. Metformin (Glucophage): 500 mg twice daily from the 3rd to the 12th week, aimed at weight control and improving insulin sensitivity, despite normal fasting glucose and glycated hemoglobin. Loprin (Aspirin): 75 mg twice daily throughout gestation. Calcium and folic acid supplementation started immediately after confirmation of pregnancy. Iron supplementation started from week 4. Arginine sachets were given in the 8th month to maintain a good uteroplacental circulation and increase amniotic fluid volume.

**Table 1:** Transvaginal ultrasound findings at 9.2 ± 1 wk of gestation.

Parameter	Value	Reference range
CRL	27.9 mm	23–30 mm
Ovary size	Rt = 3.0 × 1.8 cm Lt = 2.4 × 1.8 cm	3.5–5 cm

CRL = crown–rump length.

She remained under close antenatal surveillance. At 36 weeks, she developed gestational hypertension, which was managed with intravenous antihypertensives. A healthy male infant was delivered via elective cesarean section at 38 weeks.

In the postpartum phase, she developed left-sided breast engorgement that evolved into a small localized abscess. A minor surgical incision and drainage were performed without further complications. Three months postpartum, she initiated depot medroxyprogesterone acetate contraception (DMPA) (oral 5 mg twice daily).

The patient had a history of hypothyroidism for which she was taking Eltroxin. At 9.5 months postpartum, approximately 3 months after contraceptive initiation, she reported symptoms of thyrotoxicosis, including palpitations, weight loss, and anxiety. Eltroxin was discontinued at that time, and she was diagnosed with post-partum thyrotoxicosis. A thyroid profile revealed elevated free T3 (6.25 pg/mL), normal T4 (2.07 ng/dL), and suppressed thyroid-stimulating hormone (0.03 µIU/mL) (Table 2). Anti-thyroid peroxidase and anti-thyroglobulin antibodies were recommended but not performed due to cost constraints. She was diagnosed with postpartum-onset hyperthyroidism and treated with Neo-Mercazole (Table 3).

**Table 2:** Thyroid profile (9.5 mo postpartum, 3 mo after initiating DMPA).

Parameter	Result	Reference range
Serum-free triiodothyronine (T3)	6.25 pg/mL	2.3–4.2 pg/mL
Serum-free tetraiodothyronine (T4)	2.07 ng/dL	0.8–2.7 ng/dL
Serum thyroid-stimulating hormone (TSH)	0.03 μIU/mL	0.4–4.5 μIU/mL

DMPA = depot medroxyprogesterone acetate.

**Table 3:** Depicting the patient’s timeline.

Timeline	Age/Event	Clinical details
1st pregnancy	21 yr	- Miscarriage at 6 wk of gestation. - No hormonal or anatomical support administered.
2nd pregnancy	22 yr	- Progesterone (Cyclogest), oral dydrogesterone, and Gravibinon given. - Miscarriage at 9 wk despite support.
Post-2nd pregnancy	2 mo later	- Diagnosed with right breast fibroadenoma (2.5 cm). - Lumpectomy performed successfully.
3rd pregnancy	23 yr	- Bicornuate uterus diagnosed via transvaginal ultrasound at 9.2 wk. - Intensive medical support: • Gravibinon till fetal cardiac activity • Pubergen till 17 wk • Cyclogest till 36 wk, then reduced • Clexane till 34 wk • Metformin till 12 wk • Loprin (aspirin), calcium, and iron supplements • Arginine in 8th mo
Late pregnancy	36 wk	- Developed gestational hypertension. - Managed with IV antihypertensives.
Delivery	38 wk	- Elective cesarean section. - A healthy male infant was delivered.
Postpartum complications	Early postpartum	- Left breast abscess after engorgement - Minor incision and drainage performed.
Postpartum contraception	3 mo postpartum	- Started on DMPA (oral, 5 mg twice daily).
Postpartum thyroid dysfunction	9.5 mo postpartum	- Symptoms: palpitations, weight loss, anxiety. - Labs: ↑ FT3 (6.25 pg/mL), normal FT4, ↓ TSH (0.03 µIU/mL). - Postpartum thyrotoxicosis diagnosed. - Treated with Neo-Mercazole. - Anti-TPO and anti-Tg antibodies were not assessed due to cost.

anti-Tg = anti-thyroglobulin, anti-TPO = anti-thyroid peroxidase, DMPA = depot medroxyprogesterone acetate, FT3 = free triiodothyronine, FT4 = free thyroxine, IV = intravenous, TSH = thyroid-stimulating hormone.

The patient is clinically stable, being treated for hyperthyroidism, and is responding well to the treatment. She is still attending regular follow-ups, is clinically stable, and has developed no new complaints. This case report has been reported in line with the CARE criteria.^[4]^

## 3. Discussion

This case illustrates the multifactorial and interdisciplinary challenge of managing coexisting anatomical and endocrine disorders in a young woman with a history of recurrent pregnancy loss. The confluence of a congenital uterine anomaly, hormonally sensitive breast pathology, and delayed-onset postpartum thyroid dysfunction presents a rare and clinically instructive constellation. Despite these overlapping complications, she achieved a successful term pregnancy, underscoring the effectiveness of individualized, evidence-based, and multidisciplinary care.

Congenital uterine anomalies are reported in approximately 1 to 4% of the general population, with a bicornuate uterus comprising nearly half of these malformations. Due to abnormal fusion of the Müllerian ducts, the resulting uterine cavity compromises implantation and placental development, significantly increasing the risk of early pregnancy loss and adverse perinatal outcomes. Full-term pregnancies in such cases are relatively uncommon.^[5]^ A bicornuate uterus is linked with a 250 to 500% elevation in obstetric complications, including preterm rupture of membranes, placental abruption, cesarean delivery, and intrauterine fetal death.^[6]^ In this patient, the successful use of empirical enoxaparin therapy is noteworthy. Although thrombophilia screening was negative, low molecular weight heparin was initiated prophylactically due to the high-risk uterine architecture. The rationale aligns with case-based literature recommending empiric anticoagulation in morphologic uterine anomalies when clinical suspicion of uteroplacental insufficiency exists.

Hyperthyroidism, which developed 9.5 months postpartum in this patient, further complicated the case. Globally, it affects between 0.2% and 1.3% of the population.^[7]^ It results from excessive synthesis and release of thyroid hormones, commonly due to autoimmune or inflammatory etiologies.^[8]^ During early pregnancy, rising levels of human chorionic gonadotropin suppress thyroid-stimulating hormone, potentially delaying the recognition of subclinical thyroid dysfunction.^[9]^ In the postpartum period, immune reactivation can precipitate thyroiditis, particularly in genetically predisposed individuals. The patient’s thyrotoxicosis emerged approximately 3 months after starting depot medroxyprogesterone acetate (DMPA), suggesting a potential temporal relationship. Although thyroid autoantibodies (anti-thyroid peroxidase, anti-thyroglobulin) were not assessed due to cost limitations, the clinical profile was consistent with postpartum thyroiditis. This destructive autoimmune condition is known to present as transient hyperthyroidism or hypothyroidism within the first year following delivery.^[10]^ A similar case involving an 18-year-old postpartum woman progressing from beta-blockers to methimazole supports the clinical plausibility of this presentation.^[11]^

Additionally, this patient had a history of elevated prolactin levels and a benign right breast fibroadenoma (measuring ~2.5 cm, confirmed histologically), followed by left-sided milk congestion managed via incision and drainage. The interplay between prolactin dysregulation, thyroid dysfunction, and uterine malformation is significant. Both thyroid dysfunction and hyperprolactinemia have been independently associated with recurrent pregnancy loss. One study demonstrated a significantly increased prevalence of these disorders in women with repeated miscarriages compared to healthy controls.^[12]^

Metformin was introduced during early pregnancy to address potential insulin resistance and weight management, even in the absence of overt hyperglycemia. This off-label use is supported in cases with recurrent pregnancy loss and suspected polycystic ovarian-like metabolic profiles. Despite normal glucose and glycated hemoglobin levels, her history and weight justified a therapeutic trial.

This case reinforces the hypothesis that contraceptive-induced hormonal modulation may precipitate autoimmune thyroid disorders in predisposed individuals. Although the underlying mechanisms remain speculative, hypotheses include modulation of thyroid-binding globulin levels or immune system dysregulation induced by exogenous progestins. As this field evolves, the safety profile of long-term hormonal contraceptive use in endocrine-prone women warrants further investigation.

Overall, this case exemplifies how structured, multidisciplinary care, including early imaging, hormone modulation, empiric anticoagulation, and close postpartum monitoring, can yield favorable outcomes even in high-risk anatomical and endocrine contexts. The convergence of bicornuate uterus, breast pathology, hyperprolactinemia, and delayed postpartum hyperthyroidism has not been previously reported as a single clinical entity. This highlights the need for integrated reproductive-endocrine management and longitudinal follow-up in women presenting with complex obstetric histories.

There are some diagnostic challenges associated with Müllerian malformations. According to research, two-dimensional ultrasonography is not a practical method for diagnosing septate or bicornuate uteruses. Because X-ray hysterosalpingography and hysteroscopy do not distinguish between a septate and bicornuate uterus, they may be misdiagnosed. To differentiate among all forms of Müllerian agenesis, magnetic resonance imaging is considered the gold standard.^[13]^ Since a bicornuate uterus causes recurrent pregnancy loss, a count should be performed to determine the appropriate test for an early diagnosis.

When it comes to treating hyperthyroidism, there are several options available, including thyroidectomy, radioactive iodine therapy, and antithyroid medications. While none of these treatments is flawless, each one is sufficient to manage the condition effectively. A patient’s preferences, age, sex, underlying medical conditions, and access to specialized thyroid surgery care should all be considered when selecting a therapeutic approach. The risk of hypothyroidism must be carefully considered together with the expected treatment effects when managing hyperthyroid individuals over the long term.^[14]^

Additionally, a case report is included that showcases a successful pregnancy outcome in a patient with a bicornuate uterus, delivered through cesarean section.^[15]^ There is a study that indicates the usage of Clexane (enoxaparin) in a 23-year-old pregnant lady, even though her thrombotic symptoms were stable during pregnancy.^[16]^ The previous case report demonstrates that a postpartum lady with no prior thyroid disease exhibited symptoms of tachycardia, fever, and diarrhea; yet, lab tests revealed that she had higher thyroid levels.^[17]^

This case reflects the complex interplay between congenital uterine anomalies, recurrent pregnancy loss, endocrine dysfunction, and postpartum complications in a young woman and provides an unusual longitudinal perspective across several reproductive and endocrine issues. Although bicornuate uterus and recurrent pregnancy loss have each been reported separately, few cases report such a wide range of conditions, including benign breast disease, widespread hormonal supplementation during pregnancy, thromboprophylaxis against negative screens, postpartum thyroid failure, and contraceptive-related endocrinopathy in the same patient. Notably, this case highlights the risk of unsuspected structural abnormality in early pregnancy loss, with a need for imaging to be performed earlier in women with multiple first-trimester miscarriages. Thyroid function tests must be performed at the start of pregnancy or pre-conception, especially in women with an endocrine history. Furthermore, the emergence of postpartum thyrotoxicosis after the introduction of DMPA, while potentially coincidental, serves to raise issues regarding the relationship between thyroid autoimmunity and exogenous progestins, a field where there is currently sparse data. This case, therefore, not only contributes to the growing body of knowledge on multidisciplinary care in advanced reproductive endocrinology but also highlights under-investigated clinical associations that warrant further exploration in large cohorts.

There are a few limitations of this case report. This case details only one patient and cannot be generalized to the broader population. The diagnostic data were further limited, as hormonal and autoimmune markers could not be thoroughly evaluated due to financial constraints. Since temporal correlation between DMPA use and postpartum thyroid function decline cannot establish causation, large controlled trials are necessary.

Learning Points:

- A bicornuate uterus significantly increases miscarriage and preterm delivery risks.
- Postpartum hyperthyroidism may be unmasked or precipitated by contraceptive hormone therapy.
- Thyroid function tests must be performed at the early stage of pregnancy to diagnose any thyroid anomaly.
- Empiric Metformin and Enoxaparin may benefit high-risk anatomical pregnancies, even in the absence of lab-confirmed diabetes or thrombophilia.
- Early imaging and endocrine profiling should be routine in recurrent pregnancy loss workups.

## 4. Conclusion

This case demonstrates that a successful pregnancy is achievable in women with a bicornuate uterus and coexisting hormonal challenges through close monitoring and tailored multidisciplinary care. The use of off-label agents, such as Metformin and Clexane, although unconventional, may have contributed to a favorable outcome. Additionally, the possible association between DMPA use and postpartum thyroid dysfunction merits further study. Physicians managing women with uterine malformations should remain vigilant for subtle hormonal imbalances, and a comprehensive workup, including endocrine evaluation, should be routine in recurrent pregnancy loss. Further longitudinal studies are warranted to better understand the intersection of contraceptive use, thyroid function, and structural reproductive anomalies.

## Acknowledgments

This work has been reported in line with the CARE criteria.^[4]^ The patient had given informed consent for the case report and publication.

## Author contributions

**Data curation:** Fatima Faisal, Aiman Javed.

**Supervision:** Tirath Patel.

**Writing – original draft:** Fatima Faisal, Aiman Javed, Faisal Muzaffar, Tirath Patel, Ahmed Ibrahim, Fathimathul Henna, Bhumi Daishik Patel.

**Writing – review & editing:** Tirath Patel, Hafiza Tooba Siddiqui, Nana Sardarova, Nikhilesh Anand.

## References

[1] Alhubaishi L Alsalihi A Sharaf A Khalifa J Sharaf A. Bicornuate uterus with a rudimentary horn: management and considerations. Cureus. 2024;16:e74642. 39735105 10.7759/cureus.74642 PMC11681957

[2] Ali S Majid S Niamat Ali M Taing S El-Serehy HA Al-Misned FA. Evaluation of etiology and pregnancy outcome in recurrent miscarriage patients. Saudi J Biol Sci. 2020;27:2809–17. 32994741 10.1016/j.sjbs.2020.06.049 PMC7499272

[3] Chen S Wu F Hai R. Thyroid disease is associated with an increased risk of breast cancer: a systematic review and meta-analysis. Gland Surg. 2021;10:336–46. 33633990 10.21037/gs-20-878 PMC7882351

[4] Gagnier JJ Kienle G Altman DG Moher D Sox H Riley D; CARE Group. The CARE guidelines: consensus-based clinical case reporting guideline development. BMJ Case Rep. 2013;2013:bcr2013201554. 10.1186/1752-1947-7-223 PMC3844611 24228906

[5] Diaouga HS Laurent HL Yacouba MC. Bicornuate uterus and pregnancy: ambiguity diagnosis (a case report). Pan Afr Med J. 2022;43:203. 36942142 10.11604/pamj.2022.43.203.32905 PMC10024561

[6] Kadour Peero E Badeghiesh A Baghlaf H Dahan MH. How do bicornuate uteri alter pregnancy, intra-partum and neonatal risks? A population based study of more than three million deliveries and more than 6000 bicornuate uteri. J Perinat Med. 2023;51:305–10. 35946504 10.1515/jpm-2022-0075

[7] Wiersinga WM Poppe KG Effraimidis G. Hyperthyroidism: aetiology, pathogenesis, diagnosis, management, complications, and prognosis. Lancet Diabetes Endocrinol. 2023;11:282–98. 36848916 10.1016/S2213-8587(23)00005-0

[8] Niedziela M. Hyperthyroidism in adolescents. Endocr Connect. 2021;10:R279–92. 34596580 10.1530/EC-21-0191 PMC8558900

[9] Lee SY Pearce EN. Assessment and treatment of thyroid disorders in pregnancy and the postpartum period. Nat Rev Endocrinol. 2022;18:158–71. 34983968 10.1038/s41574-021-00604-z PMC9020832

[10] Houmøller AM Gerlif K Torp NMU Andersen SL. Diagnoses of obstetric and postpartum thyroid disease: a Danish validation study. Sci Rep. 2024;14:8777. 38627585 10.1038/s41598-024-59636-w PMC11021553

[11] Havranova J Tayel H Gallagher T Arastu M. SAT-588 unique presentation of postpartum thyroiditis with features of graves’ disease. J Endocr Soc. 2019;3(Suppl 1):SAT588.

[12] Malik P Garg Y Bedi GK Vij C. Endocrine dysfunction in recurrent pregnancy loss. Int J Contemp Med Res. 2019;6:G1–3.

[13] Passos IMPE Britto RL. Diagnosis and treatment of müllerian malformations. Taiwan J Obstet Gynecol. 2020;59:183–8. 32127135 10.1016/j.tjog.2020.01.003

[14] Hughes K Eastman C. Thyroid disease: long-term management of hyperthyroidism and hypothyroidism. Aust J Gen Pract. 2021;50:36–42. 33543160 10.31128/AJGP-09-20-5653

[15] Moltot T Lemma T Silesh M Sisay M Tsegaw B. Successful post-term pregnancy in scared bicornuate uterus: case report. BMC Pregnancy Childbirth. 2023;23:559. 37533012 10.1186/s12884-023-05875-0 PMC10394870

[16] Farquhar HE Lamprecht A. Thromboangiitis obliterans in pregnancy – case report and literature review. Obstet Med. 2022;15:68–70. 35444720 10.1177/1753495X20980251 PMC9014554

[17] Qin Y Wu Y Yu L. A case of unexpected thyroid storm in postpartum. J Int Med Res. 2025;53:3000605251335803. 40302665 10.1177/03000605251335803 PMC12046190

